# AI-Enabled Fusion of Medical Imaging, Behavioral Analysis and Other Systems for Enhanced Autism Spectrum Disorder. Comment on Jönemo et al. Evaluation of Augmentation Methods in Classifying Autism Spectrum Disorders from fMRI Data with 3D Convolutional Neural Networks. *Diagnostics* 2023, *13*, 2773

**DOI:** 10.3390/diagnostics13233545

**Published:** 2023-11-28

**Authors:** Daniele Giansanti

**Affiliations:** Centre Tisp, The Italian National Institute of Health, 00161 Rome, Italy; daniele.giansanti@iss.it; Tel.: +39-06-49902701

I am writing to you in regard to the *research article* “*Johan Jönemo, David Abramian, and Anders Eklund*—Evaluation of Augmentation Methods in Classifying Autism Spectrum Disorders from fMRI Data with 3D Convolutional Neural Networks” [1].

In the Special Issues (SIs) that I coordinate, I always try to identify an article that stimulates a discussion with scholars for wide-ranging future development. In this SI [2], I identified your article. Autism, as a neurodevelopmental disorder, affects social behavior, communication and interaction. It manifests as difficulties in understanding other people’s emotions, as verbal and non-verbal communication, as restricted interest in certain topics and as the repetitiveness of behaviors and routines. Each autistic individual is unique in his or her characteristics and level of functioning [3]. The therapeutic approach varies depending on individual needs and often involves multidisciplinary interventions.

This uniqueness is reflected in the *difficulty of diagnosis* [4,5], which requires a multifaceted approach from different medical disciplines, and in treatment that increasingly highlights the need for *personalized medicine* dedicated to autism [6,7].

Among the important activities in diagnosis are the following [4,5]:

- Observation and interviews;
- Physical exams and medical history;
- Developmental assessment and screening;
- Psychological and psychomotor evaluation;
- Assessment of social behavior and social interactions;
- Language and communication assessment;
- Sensory assessment;
- Functional behavior assessment;
- Genetic, metabolic, biochemical, immunological and neurobiological assessments;
- Assessments of environmental factors;
- Medical imaging assessment.

Functional Magnetic Resonance Imaging (fMRI) has played and is playing a significant role in advancing our understanding of autism spectrum disorder (ASD) by allowing researchers to investigate brain activity and connectivity in individuals with ASD. A search on PubMed with the composite key “*Search: (fmri [Title/Abstract]) AND (autism [Title/Abstract]) Filters: Systematic Review Sort by: Publication Date*” identified 14 systematic reviews starting from 2011, clearly identifying the potential of fMRI applied to autism, and also highlighting the need of an umbrella review [7,8,9,10,11,12,13,14,15,16,17,18,19,20,21]. fMRI allows, for example, for functional connectivity studies, Resting State fMRI (rs-fMRI), Task-Based fMRI, the neural correlation of social and communication impairments, and sensory processing and sensory integration investigation. Furthermore longitudinal fMRI studies can track brain development and changes in connectivity patterns over time in individuals with autism, which is very useful for understanding how the brain develops in those with ASD and how this leads to behavioral and cognitive changes. In fMRI, Machine Learning and Predictive Modeling have shown potential in earlier and more precise diagnosis and also in identifying specific biomarkers. Progress due to personalized interventions and therapies can be monitored just by means of fMRI, a useful tool to assess effectiveness in this filed.

I found your study very interesting and attractive.

I believe that, as you have highlighted, 3D augmentation techniques together with artificial intelligence (AI) applied to fMRI are promising.

I believe that this is a line of research that should be insisted upon.

I would also like to discuss with you the evolution prospects.

It is clear that autism requires a multidisciplinary approach and represents an area in which personalized medicine (PM) can undergo important development.

Multiple diagnostic approaches also play an important role in PM as in autism.

fMRI certainly has a key role and 3D augmentation and can represent a further boost in diagnosis and classification. AI is increasingly helping us in all of this, allowing for the classification of increasingly important volumes of data.

Personally, among the development directions of your study, I see potential in the application of AI tools to data reservoirs that, in the future, could include both the 3D-augmentation-based imaging method proposed by you and data coming from the many other multifaceted diagnostic activities not based on imaging.

I would like this comment open a scientific discussion with scholars as I am increasingly convinced that fMRI has been instrumental in uncovering the neural underpinnings of autism, shedding light on altered brain connectivity, social and sensory processing differences, and potential biomarkers; therefore, all the tools (and therefore, also the AI-based tools) integrating fMRI findings with other research approaches could contribute to a comprehensive understanding of autism and open the door to targeted interventions and treatments.
